# Prognostic value of immunohistochemical surfactant protein A expression in regenerative/hyperplastic alveolar epithelial cells in idiopathic interstitial pneumonias

**DOI:** 10.1186/1746-1596-6-25

**Published:** 2011-03-25

**Authors:** Nobuhiko Nagata, Yasuhiko Kitasato, Kentaro Wakamatsu, Masaharu Kawabata, Kazuo Fukushima, Akira Kajiki, Yoshinari Kitahara, Kentaro Watanabe

**Affiliations:** 1 Division of Clinical Pathology, Department of Clinical Research and Respiratory Medicine, National Hospital Organization Omuta Hospital, Omuta-shi, Japan; 2 Department of Respiratory Medicine, National Hospital Organization Fukuoka-Higashi Medical Center, Koga-shi, Japan; 3 Division of Respirology, Neurology, and Rheumatology, Department of Internal Medicine, Kurume University School of Medicine, Kurume-shi, Japan; 4 Department of Respiratory Medicine, National Hospital Organization Minamikyushu Hospital, Kajiki-cho, Japan; 5 Department of Respiratory Medicine, National Hospital Organization Kumamoto Saishunso Hospital, Goshi-shi, Japan; 6 Department of Respiratory Medicine, Faculty of Medicine, Fukuoka University, Fukuoka-shi, Japan

## Abstract

**Background:**

It is difficult to predict survival in patients with idiopathic pulmonary fibrosis. Recently, several proteins, such as surfactant protein (SP) and KL-6, have been reported to be useful biologic markers for prediction of prognosis for interstitial pneumonias. It is not clear whether there is any relationship between expression of these proteins in regenerative/hyperplastic alveolar epithelial cells and prognosis of idiopathic interstitial pneumonias (IIPs).

**Objectives:**

This study aimed to elucidate the clinical significance of the expression of such lung secretory proteins as SP-A and KL-6 in lung tissues of patients with IIPs.

**Methods:**

We retrospectively investigated the immunohistochemical expression of SP-A, KL-6, cytokeratin (CK), and epithelial membrane antigen (EMA) in alveolar epithelial cells in lung tissues obtained from surgical lung biopsy in 43 patients with IIPs, and analyzed the correlation between expression of these markers and the prognosis of each IIP patient. CK and EMA were used as general markers for epithelial cells.

**Results:**

In patients with usual interstitial pneumonia (UIP), the ratio of SP-A positive epithelial cells to all alveolar epithelial cells (SP-A positive ratio) in the collapsed and mural fibrosis areas varied, ranging from cases where almost all alveolar epithelial cells expressed SP-A to cases where only a few did. On the other hand, in many patients with nonspecific interstitial pneumonia (NSIP), many of the alveolar epithelial cells in the diseased areas expressed SP-A. The SP-A positive ratio was significantly lower in patients who died from progression of UIP than in patients with UIP who remained stable or deteriorated but did not die. In NSIP patients, a similar tendency was noted between the SP-A positive ratio and prognosis.

**Conclusions:**

The results suggest that the paucity of immunohistochemical SP-A expression in alveolar epithelial cells in diseased areas (i.e. regenerative/hyperplastic alveolar epithelial cells) may predict a worse prognosis for patients with IIPs, especially patients with UIP. A prospective study is needed to confirm these results.

## Background

Idiopathic pulmonary fibrosis (IPF) is a form of idiopathic interstitial pneumonia (IIP) characterized by progressive lung fibrosis and a poor prognosis. It is difficult to predict survival in patients with IPF. Longer survival has been associated with younger age, female gender, a shorter symptomatic period prior to diagnosis, less dyspnea, preserved pulmonary function, the extent of ground-glass and reticular opacities on high-resolution CT scans, lymphocytosis in broncho-alveolar lavage fluid (BALF), less fibroblastic foci in the histological sections, and a better response to treatment[1-7].

Recently, several lung secretory proteins, such as surfactant protein (SP) and KL-6, were reported to be useful biologic markers for predicting prognosis of interstitial pneumonia (IP)[8-14]. KL-6 is expressed on epithelial cells, being a mucin-like glycoprotein. Studies revealed that patients with IPF and an elevated serum level of SP-A, SP-D, or KL-6 had a poor prognosis[10-14]. On the other hand, expression of these proteins in regenerative/hyperplastic alveolar epithelium in IIP has been reported to differ among subtypes of IIP[15]. However, it is not clear whether there is any relationship between expression of lung secretory proteins in regenerative/hyperplastic alveolar epithelium and prognosis in the subtypes of IIP.

This study aimed to determine a possible relationship between patient's prognosis and immunohistochemical expression of SP-A, KL-6, cytokeratin (CK) and epithelial membrane antigen (EMA) in regenerative/hyperplastic alveolar epithelial cells of IIPs. We retrospectively examined immunohistochemical expression of SP-A, KL-6, cytokeratin (CK) and epithelial membrane antigen (EMA) in surgical lung biopsy samples obtained from 43 patients with IIP. We used CK and EMA as general markers for epithelial cells.

## Methods

### Subjects

We studied 43 patients with IIPs who underwent surgical lung biopsy between 1995 and 2006 and were followed for at least three years after biopsy in the National Hospital Organization group hospitals in Kyushu District (Omuta, Fukuoka Higashi, Minami Kyushu, and Kumamoto Saishunso Hospitals), and Kurume University Hospital. Mean follow-up time is mentioned in Table 1. The study population included 8 patients who died within two years after biopsy. Two independent pathologists reviewed the histological sections. The final IIP diagnosis incorporated histological, radiological and clinical features, and was based on the American Thoracic Society/European Respiratory Society international multidisciplinary consensus classification of the idiopathic interstitial pneumonias[16]. The study population consisted of 19 usual interstitial pneumonia (UIP) patients (13 male and 6 female, 42 to 78 years), 17 nonspecific interstitial pneumonia (NSIP) patients (11 male and 6 female, 42 to 70 years), one cryptogenic organizing pneumonia (COP) patient (female, 64 years), one desquamative interstitial pneumonia (DIP) patient (male, 59 years), one respiratory bronchiolitis-interstitial lung disease (RB-ILD) patient (male, 64 years), and four unclassifiable IP patients (three male and one female, 56 to 66 years). Table 1 summarizes the salient clinical features of the patients with UIP and NSIP, the two main subtypes of IIP in this study. Mean smoking indices were 20.4, 22.1, and 22.1 pack-years in UIP, NSIP, and unclassifiable IP patients, respectively. Smoking indices for DIP and RB-ILD patients were 90 and 44 pack-years, respectively. A COP patient was a non-smoker. No drugs were prescribed to 15 of the 19 UIP patients following biopsy. The remaining four patients were treated with 0.5 - 1.0 mg/kg/day of oral prednisolone following biopsy, which was gradually tapered. Following biopsy, 11 of the 17 NSIP patients were treated with the same dosing of oral prednisolone. Two NSIP patients were treated with 0.5 mg/kg/day of oral prednisolone and 1 mg/kg/day of oral cyclophosphamide, and one NSIP patient received 100 mg/day of oral cyclosporine A. The remaining three patients with NISP were not prescribed any drugs following lung biopsy. All DIP and COP patients were treated with 1 and 0.5 mg/kg/day of oral prednisolone, respectively, which was gradually tapered. Two patients with unclassifiable IP were treated with 10 and 15 mg/day of oral prednisolone, respectively, while no drugs were prescribed to the remaining two unclassifiable IP patients. The institutional ethical review boards of each hospital approved this study. Subjects who were alive at the time of sample collection gave written informed consent to participate in the study.

**Table 1:** Salient clinical features of patients with IIPs

	IIPs	UIP	NSIP
No. of patients	43	19	17
Age, years *	61.6 ± 8.8	63.6 ± 10.0	59.0 ± 8.6
Sex, M/F	29/14	13/6	11/6
%VC before biopsy (%)*	82.5 ± 16.6	77.8 ± 17.2	84.8 ± 17.5
PaO2 before biopsy (torr)*	77.5 ± 9.9	80.7 ± 9.8	73.7 ± 9.8
Duration of illness before biopsy (months)*	17.4 ± 21.3	13.8 ± 13.7	21.8 ± 25.1
Duration of follow up after biopsy (months)*	41.8 ± 29.6	27.3 ± 21.7	46.8 ± 21.6

* Data are presented as mean ± SD.

### Immunohistochemistry

Immunohistochemical staining was performed using the labeled streptavidin biotin method (DAKO LSAB2 kit, DAKO Japan, Kyoto). The antibodies used in this study were mouse anti-surfactant apoprotein A (SP-A) monoclonal antibody, mouse anti-cytokeratin, MNF116 (CK) monoclonal antibody, which recognizes cytokeratin 5, 6, 8, 17, and 19, and mouse anti-epithelial membrane antigen (EMA) monoclonal antibody (all obtained from DAKO Japan, Kyoto), as well as mouse anti-KL-6 monoclonal antibody (Eizai Co., Ltd., Tokyo). Endogenous peroxidase activity was blocked by treating sections with 3% H_2_O_2 _for 10 min. The sections were then sequentially treated with primary antibody (anti-SP-A, CK, EMA, or KL-6 antibody, all diluted 100×), biotinylated goat anti-mouse immunoglobulin antibody, and streptavidin-horseradish peroxidase. After these treatments, peroxidase activity was revealed by incubating the sections in 0.05% 3,3'-diaminobenzidine containing 0.001% H_2_O_2_. Sections in which the primary antibody was replaced with normal mouse IgG served as a negative control.

### Quantification of the ratio of SP-A positive epithelial cells to all alveolar epithelial cells in diseased areas (SP-A positive ratio)

The 3-micrometer thick sections stained with immunoperoxidase were examined with a microscope to which a light video camera was attached (Nikon Co., Tokyo). Images were projected on a color monitor. The total magnification was 140 (magnification of microscopic objective lens × 10 and that of the light video camera × 14). Next, images were printed out on A4-sized paper. Numbers of all alveolar epithelial cells in diseased areas, which were considered to be regenerative/hyperplastic ones, and SP-A positive epithelial cells in the same areas were counted manually on the image printouts. This allowed the calculation of the ratio of SP-A positive epithelial cells to all alveolar epithelial cells in diseased areas (i.e. regenerative/hyperplastic alveolar epithelial cells). For samples from patients with UIP, we examined areas of collapsed and mural fibrosis. Areas of end-stage lung (honeycomb) and normal lung were not included in the counting.

To determine the number of fields required to estimate the mean for each section, the number of SP-A positive epithelial cells was divided by the number of all alveolar epithelial cells in the diseased areas for five randomly selected fields in sections from 10 patients. Estimation of the SEM (standard error of the mean) within 90% confidence limits required a minimum of six fields[17]. Formal scoring was then performed in seven randomly selected fields in one section for each patient.

Using 50 randomly selected fields (10 fields from 5 patients), we assessed the variability in the number of SP-A positive epithelial cells and all alveolar epithelial cells in diseased areas counted by the two observers. The coefficients of variation were 2.1% for the number of SP-A positive epithelial cells and 3.5% for the number of all alveolar epithelial cells in diseased areas.

Immunohistochemical staining of KL-6, CK and EMA was analyzed only qualitatively.

### Assessment of fibroblastic foci (FF), established fibrosis, interstitial inflammation, and alveolar surface area in UIP

We assessed the number of FF in UIP tissue samples according to the modified method reported by Nicholson et al[18]. The 3-micrometer thick sections stained with alcian blue - periodic acid Schiff (AB-PAS) were viewed at low-power magnification (×40), and the number of FF was counted within each low-power magnification field by two observers. This was undertaken in three separate areas where activity appeared to be most marked within each biopsy, and the average FF number within a lower-power magnification field was obtained (FF score). In cases with biopsies from two different sites, an average score was taken. The coefficient of variation was 2.0%.

A semiquantitative assessment was undertaken for each individual biopsy using a scale of 0-3 to describe the extent of established fibrosis and interstitial inflammation by the two observers. For established fibrosis, a score of 0 represented no fibrosis, and a score of 3 represented end-stage or honeycomb lung. For interstitial inflammation, a score of 0 represented no inflammation, and a score of 3 represented the degree of infiltration expected in a pattern of lymphocytic interstitial pneumonia. Kappa coefficients of agreement were 0.82 for established fibrosis, and 0.46 for interstitial inflammation. Alveolar surface area was assessed quantitatively by the two observers as the ratio of alveolar surface area to the counted field using the point-counting technique. The coefficient of variation was 2.9%.

### Assessment of immunohistochemical MIB1 (Ki-67) nuclear expression in the alveolar epithelial cells in the diseased areas

To elucidate the proliferation activity of alveolar epithelial cells, we examined immunohistochemical MIB1 (Ki-67) nuclear expression in 6 UIP cases for which the SP-A positive ratios were 75.3, 64.3, 48.4, 7.7, 7.7, and 7.7%, and in 3 NSIP cases for which the SP-A positive ratios were 95.6, 80.5, and 88.6%, using the same immunohistochemical method described above and mouse anti-MIB1 (Ki-67) monoclonal antibody (DAKO Japan, Kyoto) diluted 100 ×. The number of alveolar epithelial cells staining positive for MIB1 (Ki-67) in the diseased area was expressed as a percentage of the total alveolar epithelial cells in the same areas. These were counted up to 600 cells using the same method as was used to calculate the SP-A positive ratio.

### Assessment of prognosis of the IIPs

We classified the prognosis of the IIPs into three categories: deteriorated, stable, and improved. A deteriorated prognosis was defined as follows, (1) the patient's death resulted from progression of the IIP, (2) the patient was started on long-term oxygen therapy, or (3) within the period of a year the patient's vital capacity decreased by 0.3 liters or 10%. An improved prognosis was defined as an increase in vital capacity by 0.3 liters/year or 10%/year. A stable prognosis corresponded to a disease course that could not be classified as either deteriorated or improved. The definition of acute exacerbation of IIP is based on the criteria reported by Noth and Martinez[19]. We assessed the outcome regarding the prognosis of all cases of IIP in March, 2009. The mean time following biopsy in which the assessment was done was 3.8, 3.0, 3.7, and 3.8 years in all cases, UIP, NSIP, and unclassifiable IP cases, respectively. The outcome was assessed 7.4, 12, and 4.9 years following biopsy in patients with COP, DIP, and RB-ILD, respectively.

### Statistical analysis

Data were expressed as mean ± SD (standard deviation). One-way ANOVA (analysis of variance) and Fisher's PLSD (protected least significant difference) were used to determine the correlation between the SP-A positive ratio and the prognosis of IIPs in addition to that between the prognosis of UIP and the extent of interstitial inflammation or established fibrosis, or the FF score. We evaluated the correlations between the SP-A positive ratio and percent of vital capacity (%VC), duration of illness, extent of established fibrosis, or alveolar surface area, using the Pearson's correlation coefficient. Using the unpaired t-test, we analyzed differences in the SP-A positive ratio between the patients with UIP and NSIP as well as differences between treated and untreated UIP or NSIP patients. Time differences in the follow up period after lung biopsy between patients who died from progression of UIP and stable patients were also analyzed by unpaired t-test. P < 0.05 was considered statistically significant.

## Results

### Expression of SP-A, KL-6, CK, and EMA in alveolar epithelial cells in diseased areas

**Figure 1:**
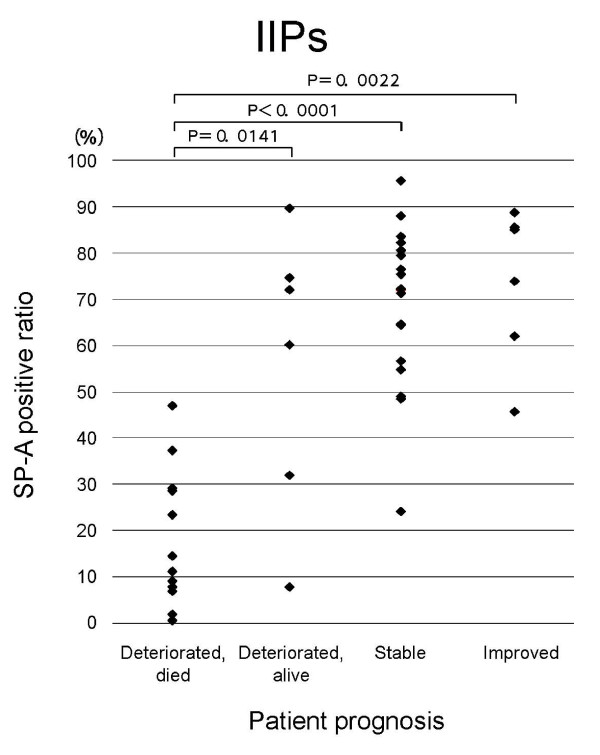
**Relation of SP-A expression in regenerative/hyperplastic alveolar epithelial cells to prognosis of IIPs in all patients**. SP-A positive ratio = number of SP-A positive epithelial cells/total number of alveolar epithelial cells in studied areas × 100.

In patients with IIP, the ratio of SP-A positive epithelial cells to all alveolar epithelial cells in diseased areas (i.e. regenerative/hyperplastic alveolar epithelial cells) varied, ranging from cases where almost all alveolar epithelial cells expressed SP-A to cases where only a few did (Figure 1). Expression patterns of SP-A in the epithelial cells of UIP patients were similar to those of IIP patients. (Figurs 2, 3A and 3B). Many alveolar epithelial cells in the diseased areas expressed SP-A in NSIP patients (Figure 4A), except for three NSIP patients in whom only a few epithelial cells expressed SP-A (Figure 5). The SP-A positive ratio was significantly lower in UIP (34.0 ± 28.1%) than in NSIP (69.1 ± 24.2%) (p < 0.0005).

**Figure 2:**
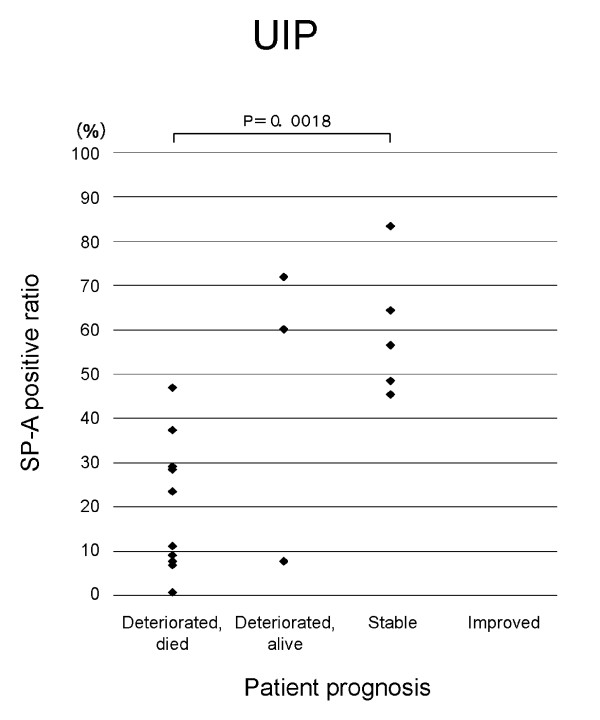
**Relation of SP-A expression in regenerative/hyperplastic alveolar epithelial cells to prognosis of UIP patients**. SP-A positive ratio = number of SP-A positive epithelial cells/total number of alveolar epithelial cells in studied areas × 100.

**Figure 3:**
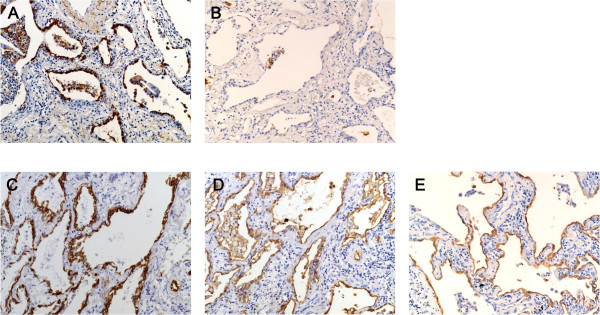
**A-E Immunohistochemical staining of SP-A (A, B), CK (C), KL-6 (D), and EMA (E) in lung tissue obtained from UIP patients**. Figs. B-E show tissue obtained from the same patient. While many regenerative/hyperplastic alveolar epithelial cells expressed SP-A in a lung tissue from a UIP patient (A), SP-A expression is observed in only a few regenerative/hyperplastic alveolar epithelial cells and in intraalveolar macrophages in lung tissue from another patient (B). Almost all regenerative/hyperplastic alveolar epithelial cells expressed CK, KL-6, and EMA (C,D,E). (Immunoperoxidase and hematoxylin, × 140).

**Figure 4:**
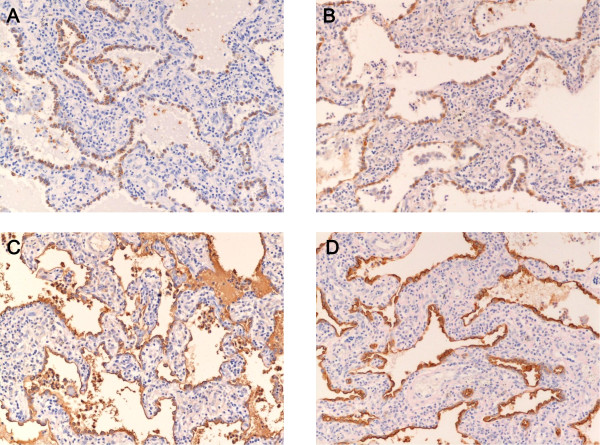
**A-D Immunohistochemical staining of SP-A (A,), CK (B), KL-6 (C), and EMA (D) in lung tissue obtained from an NSIP patient**. Many regenerative/hyperplastic alveolar epithelial cells expressed SP-A, CK, KL-6, and EMA in lung tissue from an NSIP patient. (Immunoperoxidase and hematoxylin, × 140).

**Figure 5:**
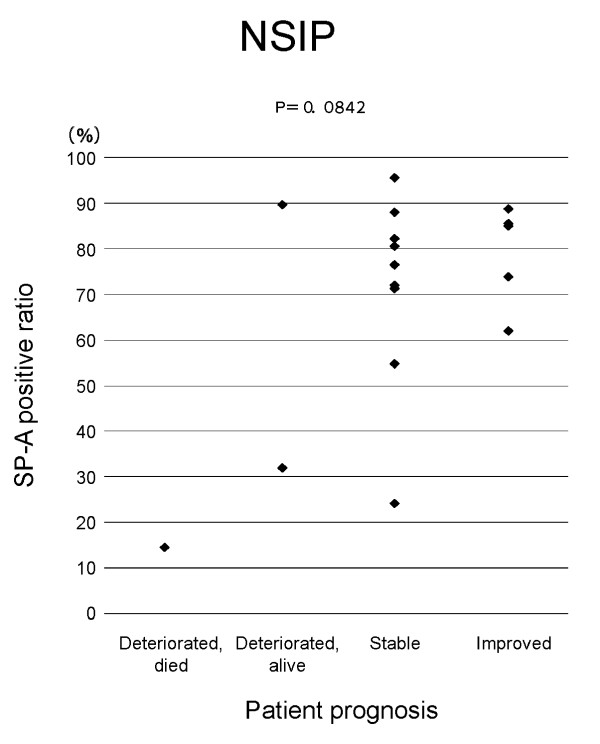
**Relation of SP-A expression in regenerative/hyperplastic alveolar epithelial cells to prognosis of NSIP patients**. SP-A positive ratio = number of SP-A positive epithelial cells/total number of alveolar epithelial cells in studied areas × 100.

Almost all alveolar epithelial cells in diseased areas revealed CK immunoreactivity (Figures 3C and 4B) in patients from all IIP groups, with the exception of three patients (one patient each with NSIP, RB-ILD, and unclassifiable IP) in whom only a few epithelial cells expressed CK.

In all IIP patients, almost all alveolar epithelial cells in diseased areas stained positive for KL-6 (Figures. 3D and 4C) and EMA (Figures. 3E and 4D).

### Prognosis of IIPs

Table 2 lists the prognosis of all IIP patients as well as the prognosis for patients of each subtype (UIP, NSIP, COP, DIP, RB-ILD, and unclassifiable IP).

**Table 2:** Prognosis of IIP patients

	IIPs	UIP	NSIP	COP	DIP	RB-ILD	unclassifiable
Number of patients	43	19	17	1	1	1	4
Prognosis (number of patients)							
deteriorated, died (died of acute exacerbation)	13 (12)	11 (10)	1 (1)	0	0	0	1 (1)
deteriorated, alive	6	3	2	0	0	0	1
stable	18	5	9	0	1	1	2
improved	6	0	5	1	0	0	0

### Correlation between immunohistochemical expression of SP-A, KL-6, CK and EMA in alveolar epithelial cells in diseased areas and prognosis of IIPs

In patients with IIPs as a whole and in those with UIP, the SP-A positive ratio was significantly lower in those who died from progression of IP than in patients with another prognosis (stable, improved and deteriorated but alive) (Figs. 1 and 2). The SP-A positive ratio for 10 out of 11 UIP patients who died from progression of UIP was less than 0.4. On the other hand, the SP-A positive ratio for only one out of 8 UIP patients who showed another prognosis was less than 0.4. In NSIP patients, a similar trend was noted for the SP-A positive ratio in alveolar epithelial cells in diseased areas and prognosis (p = 0.0842) (Figure 5). In IIP patients, no specific relationship was apparent between immunohistochemical expression of CK, KL-6, or EMA in the alveolar epithelial cells in diseased areas and prognosis of IIPs.

### Correlation between the FF score, extent of established fibrosis, or interstitial inflammation and UIP prognosis

**Figure 6:**
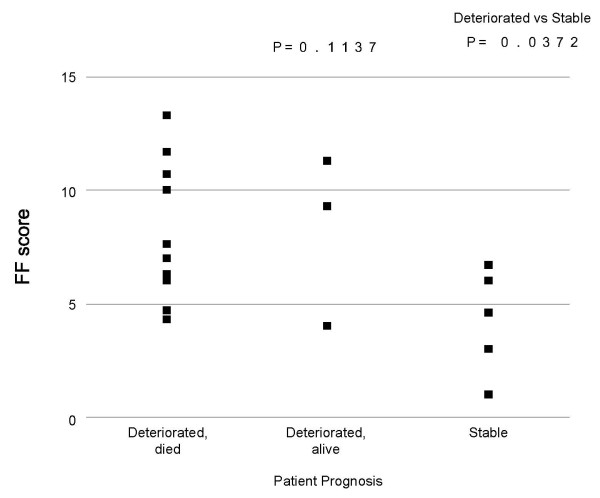
**Correlation between FF score and UIP prognosis**. The FF score indicates the number of fibroblastic foci within a lower-power magnification field.

No significant relation was seen between the FF score and each prognosis of UIP, that is, stable, deteriorated but alive, and deteriorated and died (p = 0.1137), though the FF score was significantly higher in patients who showed deteriorated clinical course than in those with stable clinical course (p = 0.0372, Figure 6). No specific correlation was apparent between UIP prognosis and either the extent of established fibrosis (p = 0.4287), or interstitial inflammation (p = 0.1554).

### Correlation between the SP-A positive ratio and FF score, the extent of established fibrosis, or alveolar surface area in UIP

**Figure 7:**
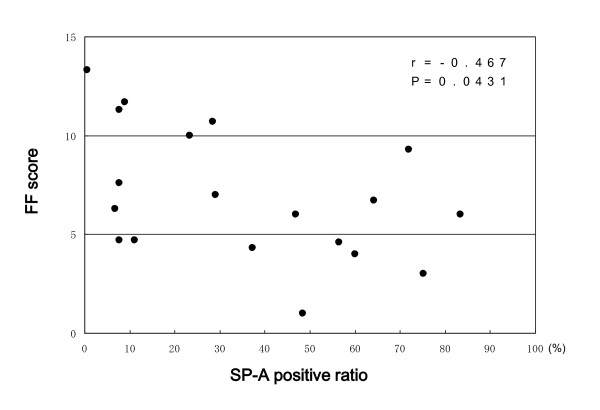
**Correlation between the SP-A positive ratio and FF score in UIP patients**. SP-A positive ratio = number of SP-A positive epithelial cells/total number of alveolar epithelial cells in studied areas × 100. FF scores indicate the number of fibroblastic foci within a lower-power magnification field.

There was a significant inverse correlation between the SP-A positive ratio and the FF score (r = -0.467, p = 0.0431, Figure 7). There was no correlation between the SP-A positive ratio and either the extent of established fibrosis (r = 0.101, p = 0.7041) or alveolar surface area (r = -0.217, p = 0.5886).

### Immunohistochemical MIB (Ki-67) nuclear expression in the alveolar epithelial cells in diseased areas

Cells staining positive for MIB (Ki-67) were observed in 1.8, 3.7, 1.3, 1.9, 1.7, and 2.7% of all alveolar epithelial cells in collapsed and mural fibrosis areas in UIP cases for which the SP-A positive ratios were 75.3, 64.3, 48.4, 7.7, 7.7, and 7.7%, respectively. In NSIP cases for which the SP-A positive ratios were 95.6, 88.6, and 80.5%, cells staining positive for MIB (Ki-67) were observed in 4.5, 7.6, and 5.8%, respectively, of all alveolar epithelial cells in diseased areas.

### SP-A positive ratio between treated and untreated UIP or NSIP patients

The mean SP-A positive ratio of treated and untreated UIP patients was 41.8% and 33.9%, respectively, and that of treated and untreated NSIP patients was 71.4% and 61.7%, respectively. There was no statistically significant difference in the SP-A positive ratio between treated and untreated UIP (p = 0.6474) or NSIP patients (p = 0.4987).

### Follow-up periods after lung biopsy in UIP patients who died from progression of UIP, and who showed stable clinical course

The five UIP patients with a stable clinical course after lung biopsy were followed for a significantly longer period (39.0 ± 15.6 months) than the 11 patients who died from progression of UIP (17.2 ± 12.2 months) (p = 0.0071).

### Correlation between the SP-A positive ratio and illness duration prior to lung biopsy, and %VC at the time of lung biopsy

Thirty-three patients visited hospitals because of subjective symptoms. The mean SP-A positive ratio for this patient group was 53.7 ± 29.3. There was no correlation between the SP-A positive ratio and %VC at the time of lung biopsy for any of the patients (r = 0.023, p = 0.8860), or in those who visited hospitals because of subjective symptoms (r = 0.101, p = 0.5853). Similarly, no correlation was found between the SP-A positive ratio and duration of illness before lung biopsy in all patients (r = -0.065, p = 0.6886), or in those who visited hospitals because of subjective symptoms (r = -0.044, p = 0.8117).

## Discussion

This is the first report to describe the relationship between immunohistochemical SP-A expression in alveolar epithelial cells in diseased areas (i.e., regenerative/hyperplastic alveolar epithelial cells) and prognosis of IIPs, especially UIP. From the present study, a paucity of immunohistochemical SP-A expression in regenerative/hyperplastic alveolar epithelial cells is considered to predict worse prognosis of IIPs, especially UIP. The presence of significant inverse correlation between the SP-A positive ratio and the FF score, which has already been reported to predict the prognosis of UIP[18], also supports this result. It is possible that UIP patients whose regenerative/hyperplastic alveolar epithelial cells did not express SP-A had a long-standing illness preceding lung biopsy, and therefore showed poor prognosis. However, there was no correlation between the SP-A positive ratio and the duration of illness or the %VC at the time of lung biopsy. Therefore, we consider this possibility highly unlikely.

We anticipate that more UIP patients might die if they are observed for a longer period. Though we conducted this study retrospectively, UIP patients with stable clinical courses were observed for significantly longer periods compared to those who died from UIP progression. Therefore, it is unlikely that differences in the follow-up period after lung biopsy affected the assessment of prognosis.

Regenerative/hyperplastic alveolar epithelial cells that did not express SP-A expressed other markers such as CK, KL-6, and EMA. Among the cells that did not express SP-A, a few regenerative/hyperplastic alveolar epithelial cells expressing SP-A were intermingled. In addition, type II alveolar epithelial cells seen in the normal alveolar tissue among the diseased lung tissue were stained positively with SP-A. From these findings, we think that the lack of SP-A expression was not an artifact, and did not result from inappropriate fixation or staining techniques.

In a preliminary study, we found that only a few epithelial cells lining the honeycomb expressed SP-A, irrespective of SP-A positive ratio of collapsed and mural fibrosis areas in UIP. Other investigator also reported the paucity of SP-A positive epithelial cells in the honeycomb lesion[15]. Counting results are thus highly influenced by the honeycomb area, and might only reflect the occupied area of honeycomb lung, provided the area is included in the counting. Therefore, we did not include the honeycomb area in the counting. On the other hand, we did not also include normal area in the counting, because the aim of the present study was to investigate the expression of epithelial cell markers such as SP-A in regenerative/hyperplastic alveolar epithelial cells of IIPs.

We examined how corticosteroid or immunosuppressive therapy might affect expression of SP-A in alveolar epithelial cells and found no statistically significant differences in the SP-A positive ratios between treated and untreated UIP or NSIP patients. Therefore, it is unlikely that corticosteroid or immunosuppressive therapy affected the correlation between the SP-A positive ratio and the prognosis for UIP or NSIP patients, although the patient population was small.

The reason a paucity of immunohistochemical SP-A expression related to a worse prognosis in UIP patients is unclear. SP-A is reported to play an important role in the regulation of pulmonary inflammation[20-23]. Therefore, a lack of SP-A expression may induce an exaggerated inflammatory reaction, such as an overproduction of inflammatory cytokines to a given stimulus (a viral infection, for example) and cause deterioration of UIP. We do not know whether the regenerative/hyperplastic alveolar epithelial cells that lacked SP-A expressed other surfactant apoproteins. If expression of surfactant as a whole is poor in lungs of UIP patients, the patients may suffer from a condition similar to respiratory distress syndrome, for which the clinical course is poor. The type II alveolar epithelial cell is the major source of pulmonary surfactant, which functions to reduce surface tension forces and the tendency of the alveolar spaces to collapse. The reduction in surfactant production by the type II cells in IPF[23-26] may promote atelectasis, resulting in the apposition and fibrotic fusion of the denuded alveolar basement membranes[27]. Alveolar epithelial markers, which reflect the capacity of the type II cells to respond appropriately to injury, may predict clinical outcome of IPF.

McCormac et al. reported that IPF patients whose BALF showed a low SP-A to total phospholipid ratio experienced a worse prognosis[8,9]. Though we did not measure serum or BALF SP-A concentrations in the present study, our current results are consistent with those of McCormac et al. Previous studies report that IPF patients with high SP-A serum concentrations had poor prognoses[10-12]. Takahashi et al. reported a time lag between SP-A or D concentrations in the serum and BALF in radiation-induced lung injury in rats[14]. This may explain the different results reported for the correlation between SP-A or D concentrations in the serum or BALF and IPF prognosis.

The SP-A positive ratio in lung tissue of UIP patients was significantly lower than that for NSIP patients. This difference may reflect the different courses of disease progression in these two types of interstitial pneumonia. Another possible explanation is that characteristics or function of regenerative epithelial cells differ between UIP and NSIP. Though the number of samples studied was small, the different percentages of alveolar epithelial cells staining positive for MIB1 (Ki-67) between UIP and NSIP patients noted in the present study may support this idea. In an investigation of the phenotypes of regenerative epithelial cells in UIP and NSIP patients, Hinata et al. proposed different origins of regenerative epithelial cells in the two interstitial pneumonias[15].

As the present study was retrospective, a prospective approach is necessary to confirm the relationship between immunohistochemical expression of surfactant protein to the patient prognosis in IIPs, particularly UIP.

## Conclusions

The results of the present study suggest that the paucity of immunohistochemical SP-A expression in regenerative/hyperplastic alveolar epithelial cells may predict a worse prognosis for patients with IIPs, especially those with UIP. A prospective study is needed to confirm these results.

## Competing interests

The authors declare that they have no competing interests.

## Authors' contributions

NN initiated the investigation, developed the study design, managed the investigation, and drafted the paper. YK, KW, MK, KF, AK and YK managed data collection. KW managed the investigation. All authors read and approved the manuscript.
